# A new approach for Small Ruminant Lentivirus full genome characterization revealed the circulation of divergent strains

**DOI:** 10.1371/journal.pone.0212585

**Published:** 2019-02-21

**Authors:** Barbara Colitti, Elisabetta Coradduzza, Giantonella Puggioni, Maria Teresa Capucchio, Ramsés Reina, Luigi Bertolotti, Sergio Rosati

**Affiliations:** 1 University of Turin, Dept. Veterinary Science, Grugliasco, Torino, Italy; 2 Istituto Zooprofilattico Sperimentale della Sardegna, Sassari, Italy; 3 Institute of Agrobiotechnology (CSIC-UPNA-Government of Navarra), Navarra, Spain; Boston College, UNITED STATES

## Abstract

Small Ruminant Lentiviruses (SRLV) include at least 4 viral highly divergent genotypes. Genotypes A and B are widely distributed and genotypes C and E have been recognized in restricted geographic areas. New phylogroups have been identified targeting conserved regions. However, this approach suffers from the potential risk to misamplify highly divergent strains. Pathogenic strains are easily adapted to fibroblastic cells, but non-pathogenic strains isolation may require a different approach. We developed a fast and effective method for SRLV full genome characterization after cell culture isolation. Spleen samples were collected during regular slaughter from sheep and goats in northwestern Italy. Spleen-derived macrophage cultures were monitored for reverse transcriptase activity and RNA was extracted from the supernatant of positive cultures. Using Illumina MiSeq platform 22 new full genome sequences were obtained. The success of this approach is based on the following features: spleen is one of the main target for SRLV persistence; red pulp is a reserve of resident macrophages, the main target for SRLV replication in vivo; RTA is a sensitive assay for any replicating retrovirus; de novo sequencing do not require genetic knowledge in advance.

## Introduction

Small Ruminant Lentiviruses (SRLV) include, to date, 4 highly divergent viral genotypes. The genetic differences among viral strains are related to antigenic and biological properties both *in vitro* and *in vivo*. Historically, Visna Maedi virus (MVV) and Caprine Arthritis Encephalitis virus (CAEV) were first isolated from sheep and goat respectively, and have been considered for long time to be strictly associated to specific clinical features and host. While those viruses are still considered prototypes of the widely distributed genotypes A and B, a number of sub-genotypes, within group A and B, and new genotypes (C and E) have been recognized. Consequently, SRLV are now considered host adapted but not strictly host associated. Moreover, the differences defined in the past [1] are becoming less clear due to the increasing number of available sequences.

Pathogenic strains are frequently isolated from specific or suggestive gross lesions by tissue explantation (i.e. lung, udder, synovial membrane) or co-cultured with permissive cells, typically fibroblast-like cells. Strains that achieve adaptation to fibroblastic cells, overgrow and show the typical cytopathic effect characterized by syncitia formation. In the early nineties, through the viral isolation method, a number of sheep and goat pathogenic strains were isolated and later characterized as B2 and B1 subtype respectively [2,3]. Unfortunately this approach may potentially fail in the detection of new genotypes (i.e. unsuccessful isolation or unclear cytopathic effect) due to the limited capacity of some low pathogenic strains to adapt to fibroblasts [4]. In order to overcome this problem, molecular approaches based on new PCR protocols were developed. These tools strongly support the SRLVs characterization, increasing the knowledge about their genetic heterogeneity [4]. This was the case of the genotype E: biological characterization *in vitro* and *in vivo* of the subtype E1, known as *Roccaverano* strain, opened new insights into putative non-pathogenic strains, being able to grow productively only in macrophage culture and including their role in mitigating the pathogenic potential of more virulent strains [5,6].

With the advent of the Next Generation Sequencing (NGS) technology, new opportunities became available to fully characterize SRLV isolates, even in the absence of previous knowledge of genetic and biological properties.

Keeping in mind that low pathogenic SRLVs may have a restricted cell tropism and may be difficult to isolate from standard tissue explantation, we developed a fast and effective method for SRLV full genome characterization after cell culture isolation. Spleen explant cultures were performed from goats and sheep sampled during slaughtering and NGS protocols from reverse transcriptase activity positive cultures were applied. By this approach, 22 full genomes were assembled representing two major genotypes. In addition, beside the pathogenic B1 and B2 subtypes, a large number of isolates belonging to the subtype A8 was found.

## Material and methods

### Sample collection and virus isolation

Blood and spleen paired samples were collected from adult ovine and caprine local breeds. Two slaughterhouses in the Piedmont Region, Northwestern Italy, were chosen in order to cover different geographical areas and to increase the variability of samples. Animals were collected randomly during the standard slaughtering activities. (see details in Table 1)

**Table 1:** Samples characterized in the present study.

Isolate (Accession num)	Host	Elisa Screening	Elisa Genotying	CPE	RT activity (passage)	Gag subtype
To1_89 (MH374290)	goat	na	na	yes	1	B1
Taccone (MH374289)	goat	na	na	yes	1	B1
VdA (MH374291)	goat	Positive	A	no	1	A8
It001.2017 (MG554402)	sheep	Positive	Indet	yes	4	B2
It002.2017 (MG554403)	goat	Positive	Indet	no	1	A8
It003.2017 (MG554404)	goat	Positive	A	no	1	A8
It004.2017 (MG554405)	goat	Positive	A	no	1	A8
It005.2017 (MG554406)	goat	Positive	Indet	no	1	A8
It006.2017 (MG554407)	goat	Positive	E	yes	1	A8
It007.2017 (MG554408)	goat	Positive	Indet	no	1	A8
It009.2017 (MG554409)	goat	Positive	A	yes	1	A18 (A3-A4)
It010.2017 (MG554410)	goat	Positive	B	yes	1	B1
It014.2017 (MG554411)	goat	Positive	Indet	yes	1	B1
It016.2017 (MG554412)	goat	Positive	B	yes	1	B1
It017.2017 (MG554413)	goat	Positive	Indet	yes	1	B1
It020.2017 (MG554414)	goat	Positive	B	yes	1	B1
It024.2017 (MH374283)	goat	Positive	A	yes	3	A8
It025.2017 (MH374284)	goat	Positive	A	no	1	A8
It026.2017 (MH374285)	goat	Positive	A	no	1	A8
It032.2017 (MH374286)	goat	Negative	Negative	no	3	A8
It038.2017 (MH374287)	sheep	Positive	A	yes	3	A19 (A9-11)
It042.2017 (MH374288)	sheep	Positive	Indet	yes	3	B2

Blood serum was used for antibody screening and genotyping using a commercially available ELISA kit (Eradikit—SRLV Starter kit, In3diagnostic, Italy); spleen was promptly delivered to the laboratory for tissue explantation. Briefly, the splenic capsule was disinfected with 70% ethanol and a 5 ml syringe with G21 needle was inserted into the splenic pulp. A negative pressure was applied by the syringe plunger while the needle was guided in different directions into the splenic tissue. When enough material was extracted into the nozzle, the syringe was removed and pulp material resuspended into 5ml of DMEM supplemented with L-glutamine 1mM and 2X antibiotic/antimycotic solution (Sigma Aldrich). During the second sampling period the described preliminary procedure was accomplished directly at the slaughterhouse. After 4h incubation at 37°C, medium was removed and tissue fragments were seeded on 25cm^2^ flasks in complete macrophage medium consisting in RPMI medium supplemented with L-glutamine 2mM, 1% non-essential amino acids, vitamins, sodium pyruvate 1mM, 2-mercaptoethanol 17μM, gentamicin 50μg/ml and FBS 10%. Cultures were maintained at 37°C in a humidified atmosphere containing 5% CO_2_ and medium was partially replaced every 3–7 days.

Once a week, medium was collected and reverse transcriptase activity was determined using Lenti RT activity kit (Cavidi, Uppsala, Sweden). RT activity was tested and recorded for each culture passage, in order to evaluate its trend. In the case of RT activity positive outcome, all medium was collected and further processed for whole genome sequencing. The presence of cytopathic effect in fibroblastic cells was recorded at each culture passage. In the presence of fibroblastic overgrowth, trypsin sensitive cells were periodically removed from the original flask.

### MiSeq run

RT positive cell culture supernatants were centrifuged for 20 minutes at 600 g to eliminate cell debris and were concentrated with Amicon-15 100 kDa centrifugal filter tubes (Millipore Merck KGaA, Darmstadt, Germany).

Viral RNA was extracted with QiAmp Viral RNA Kit (Qiagen, Hilden, Germany) and quantified using Nanodrop system (Thermo Fisher Scientific). Viral RNA was reverse transcribed into double stranded cDNA with Maxima H Minus Double–stranded cDNA Synthesis kit (Thermo Fisher Scientific) in accordance with manufacturer instructions and quantified with a fluorimetric method, Qubit dsDNA kit (Life Technologies). Samples were used for DNA library preparation using the Nextera XT DNA Library Prep Kit (Illumina, San Diego, CA, USA), according to the manufacturer’s protocol. The quantity of DNA was assessed using Agilent DNA High Sensitivity chip assay (Agilent Technologies) and the Qubit dsDNA kit (Life Technologies). Paired-end libraries were sequenced using Illumina V2 chemistry and Illumina MiSeq platform.

### Data analysis

Reads obtained by the MiSeq runs were checked for quality (FastQC) and trimmed (Trimmomatic ver. 0.32). Two parallels pipelines were followed. Reads were aligned to all known reference genomes in order to identify and confirm the viral genotype (Geneious ver. 11.1.2); the reads were further aligned to the consensus sequence obtained after the first step, in order to confirm the genome sequence. In parallel, the reads were used for *de novo* assembling (Velvet software ver. 1.2.10); the obtained contigs were compared to the consensus sequence derived from resequencing. Annotation of the main genes was performed manually, by comparing amino acidic sequences among new and reference genomes, as well as the LTR regions. Genotype was determined basing on the Gag gene sequence alignment as previously reported [1,6]. *Gag* gene and the complete genome sequence were used to depict phylogenetic relationships between the newly characterized and the reference strains using a Bayesian approach implemented in MrBayes package [7]. Basing on the phylogenetic tree topologies, the association between genetic sequence features and RT activity and CPE was calculated using the algorithms implemented in Bats software ver. 0.9 based on Bayesian Markov-Chain Monte Carlo approach to the investigation of phylogeny–trait correlations [8].

## Results

A total of 42 paired samples (spleen and blood serum) were collected from 16 sheep and 26 goats. Thirty-three were antibody positive and 26 were serotyped according to the reactivity against an immunodominant linear epitope of the capsid antigen, able to discriminate among genotype A, B, and E [2,9,10].

Twenty-six RT activity positive cultures from spleen explants were obtained (25 from seropositive and 1 from a seronegative animal) and further processed for whole genome sequencing. Nineteen isolates were readily available after the first collection time (about 10 days post-culture) or after the first passage (after 17 days of culture), while additional 7 isolates showed RT activity after 3–4 weeks of culture. Interestingly, only half (13 out of 26) of the strains showed cytopathic effect (CPE) on overgrowing fibroblastic-like cells, characterized by typical cell fusion.

Considering a coverage cut-off of 100x, full genome sequence were obtained from 22 isolates out of 26, using the proposed method. Four samples which did not meet the coverage criteria were not taken into account. Sequence analysis revealed the presence of genotypes A and B. The heterogeneity within each genotype was quite high, confirming the circulation of subtypes A8, B1 and B2, based on the similarity of *gag* genes. Moreover, one sample from sheep (It038.2017) showed a large difference with known sequences (at least around the 24%) belonging to the same monophyletic clade together with A9 and A11 subtypes. In the same way, the isolate It009.2017 was genetically related to subtypes A1 and A4 but differences were within the 25%-15%. Following the criteria published before [1], these samples suggest the presence of new subtypes (A18 and A19) and confirm the very high heterogeneity of VMV-like viral strains.

As reported before, genotypes A and subtype B1 were identified in samples from goats and sheep, whereas B2 was only found in ovine samples. Sequences obtained from animals belonging to the same flock clustered together, suggesting a clonal origin of the viral strain.

No differences in terms of SRLV positivity or genotyping between the two slaughterhouses were recorded. A positive association was observed between the phylogroup A8 and the absence of CPE in culture: the observed Monophyletic clade value mean was 5.015 (p < 0.10) and indicated a significant correlation between in vitro features and phylogenetic relationship among A8 new strains. The relevant data are summarized in Table 1. All but one serum sample were correctly classified using the serotyping ELISA according to the paired strain sequence analysis, while a single sample (It006.2017) gave spurious results (E in serotyping vs A8 subtype in sequencing). Sequence analysis of the *gag* gene encompassing the immunodominant epitope revealed a single non-synonymous mutation P231Q, a specific signature of genotype E [4] (Table 2). On the other hand HV1 and HV2 regions along env gene sequence did not show motifs that can be clearly associated to a single subtype or to low pathogenic features (Table 3).

**Table 2:** Alignment of the immunodominant epitope within the gag gene. Reference genotype are reported in bold. Dots indicate identical residue comparing to the reference K1514 (MVVlike A genotype).

Isolate	Subtype	Capsid epitope
**K1514**	**A1**	**QKELIQGKLNEEAERWVRQNPPGP--NVLTVDQ**
It025.2917	A8	. . . . . . . . . . . . . . . . . . . . . . . .--. . . . . . .
It007.2017	A8	. . . . . . . . . . . . . . . . . . . . . . . .--. . . . . . .
It005.2017	A8	. . . . . . . . . . . . . . . . . . . . . . . .--. . . . . . .
ItVdA.2017	A8	. . . . . . . . . . . . . . . . . . . . . . . .--. . . . . . .
It038.2017	*A18*	. . . . . . . . . . . . . . . . . . . . . . . .--. . . . . . .
It009.2017	*A19*	. . . . . . . . . . . . . . . .I. . . . . . .--. . . . . . .
It024.2017	A8	. . . . . . . . . . . . . . . . . . . . . . . .--Q. . . . . .
It026.2017	A8	. . . . . . . . . . . . . . . . . . . . . . . .--Q. . . . . .
It004.2017	A8	. . . . . . . . . . . . . . . . . . . . . . .Q--. . . . . . .
It002.2017	A8	. . . . . . . . . . . . . . . .I. . . . . . .--. . . . . . .
It003.2017	A8	. . . . . . . . . . . . . . . .M. . . . . .Q--. . . . . . .
It006.2017	A8	. . . . . . . . . . . . . . . .M. . . .Q----.A. . . . .
**Cork**	**B1**	**. . . . . . . . . . . . . . . .R.N . . .P.AGGG. . . . .**
It016.2017	B1	. . . . . . . . . . . . . . . .R.N . . .P.AGGG. . . . .
It010.2017	B1	. . . . . . . . . . . . . . . .R.N . . .P.QGGG. . . . .
It020.2017	B1	. . . . . . . . . . . . . . . .R.N . . .PQAGGG. . . . .
It017.2017	B1	. . . . . . . . . . . . . . . .R.N . . .PQGGGG. . . .L
It014.2017	B1	. . . . . . . . . . . . . .Q.R.N . . .PQAGGA. . . . .
It001.2017	B2	. . . . . . . . . . . . . . . .R.N . . .PQAGGG. . . . .
It042.2017	B2	. . . . . . . . . . . . . . . .R.N . . .PQAGGG. . . . .
**EU010124 Roccaverano**	**E1**	**V . . .V.D . . .K . . .T.M. . . .QP.--GG. . . . .**

**Table 3:** Variable regions within env gene among SRLV A genotype strains. HV1 and HV2: hypervariable (HV) region; TM: transmembrane domain within env.

ENVELOPE REGIONS	HV1	HV2	V5	TM
VLVLV1A K1514	VGNGTITGNCSVTNWDG	NKWTCAARRK--GSRRDSLYIAG-RD	QSYMEAQGENRRS	ELDCWHYQHYCVTS
VLVLV1B K1514	. . . . . . . . . . . . . . . . .	. . . . . . . .TGRK. .Q. . . . . . . .-. .	. . . . . . . .K. . . .	. . . . . . . . . . . . . .
NC 001452 kv1772	. . . . . . . . . . . . . . . . .	. . . . .------K.Q . . .-. . . .-. .	. . . . . . . . . .K. .	. . . . . . . . . . . . . .
VLVGAGA_kv1772	. . . . . . . . . . . . . . . . .	. . . . .------K.Q . . .-. . . .-. .	. . . . . . . . . .K. .	. . . . . . . . . . . . . .
VLVCG_Visna/Maedi	. . . . . . . . . . . . . . . . .	. . . . .------K.Q . . .-. . . .-. .	. . . . .ER. . . . . .	. . . . . . . . . . . . . .
VLVCGA_LV1.1	. . . . . . . . . . . . . . . . .	. . . . .------K.Q . . .-. . . .-. .	. . . . .ER. . . . . .	. . . . . . . . . . . . . .
**It038.2017**	**VGNGTITGNCSVTNWDG**	**. . . . . .P.WGKG. .--. . . . . . .G.Q**	**DQ.LKTNKRRK. .**	**. . . . . . .H.F. . . .**
AF479638_P1OLV	. . . . .L. . . . . . .D . . .	RQ . . .S. .VG--.TT. . . . . . . .-.N	KA.S.KKKRQPQ-	. . . . . . . . . . . . . .
OLVCG_SAOMVV	. . . . . . . . . . . . .D.E.	. . . . . . . .NS--KKK. . . . . . . .-. .	KA.R.KNMR.K. .	. . . . . . . . . . . . . .
NC_001511_Ovinelentivirus	. . . . . . . . . . . . .D.E.	. . . . . . . .NS--KKK. . . . . . . .-. .	KA.R.KNMR.K. .	. . . . . . . . . . . . . .
**It009.2017**	**. . . . . . . . . . . . .D . . .**	**K. . . . . . .AN--DK. . . . . . . . .-.N**	**N. . . .KNRKKQKR**	**Q. . . . . . . . . . . . .**
KT453988_g6221	I. . . . . . . . . . . . . . . .	.Q. . . . . .TA--.KK. . . . . . . .-. .	. . . .QN.EK.K.A	. . . . . .H. .F. . . .
KT453990_s7631	I. . . . . . . . . . . . . . . .	.Q. . . . . .TA--.E. . . . . . . . .-. .	. . . .QN.EK.K.A	. . . . . .H. .F. . . .
KT453989_s7385	I. . . . . . . . . . . . . . . .	.Q. . . . . .TA--.KK. . . . . . . .-. .	. . . .QN.EK.K.A	. . . . . .H. .F. . . .
HQ848062_Ov697	. . . . .V. . . . . . . . . . .	.T . . .S. .K.--.-. . . . . . . . .-. .	. . .I.S.EK.K. .	. . . . . .H. .F. . . .
KY358788_USMARC-199906011-2	. . . . . . . . . . .A. . . . .	.Q . . .K . . .S--.NKT. . . . . . .-GE	. . . . .T.RRKK. .	. . . . . .H. .F. . . .
KY358787_USMARC-200303013-1	. . . . . . . . . . . .K. . . .	.L. . . .P. .R--.NVT. . . . . . .-GK	K. .I.T.RRKK.A	. . . . . .H. .F. . . .
**ItVdA.2017**	**. . .D. . . . . . . . .D . . .**	**.E. . . . . .QR--NDK. . . . . . . .-.N**	**T. .VDQKRGKKKR**	**. . . . . . . . .F. . . .**
**It024.2017**	**. . . . . . . . . . . . . . . . .**	**. . . . . . . .KEKGKGQQ. . . . . . .-. .**	**T. .V.QS.KSKNR**	**. . . . . . . . .F. . . .**
**It026.2017**	**. . . . . . . . . . . . . . . . .**	**. . . . . . . .K.--KGQQ. . . . . . .-. .**	**T. .V.QS.K.KNR**	**. . . . . . . . .F. . . .**
**It007.2017**	**. . . . . . . . . . . . .D . . .**	**.M. . . . . .QS--.E. . . . . . . . .-.N**	**TN.V.L.KRRQKR**	**. . . . . . . . .F. . . .**
**It006.2017**	**. . . . . . . . . . . . . . . . .**	**G. . . . . . .Q.--RDKQ. . . . . . .-.N**	**T. .V.QN.KKKKR**	**. . . . . . . . .F. . . .**
**It005.2017**	**. . .N. . . . . . . . .D . . .**	**. . . . . . . .KQ--EEQW. . . . . . .-.N**	**T. .I.Q.KGKKKR**	**. . . . . . . . .F. . . .**
**It002.2017**	**K. . . . . . .A.N. . . . . .**	**.R. . . . . .Q.--DG. . . . . . . . .-.N**	**T. . . .QTRGKKKR**	**. . . . . . . . .F. . . .**
**It025.2017**	**. . .N. . . . . . . . . . . . .**	**D. . . . . . .QN--NEAQ. . . . . . .-.N**	**TA.V.Q.-KRKKR**	**. . . . . . . . .F. . . .**
**It003.2017**	**A. .N. . . . . . . . .D . . .**	**S. . . . .E.Q.--ENKT. .V. . . .-.E**	**T. .V.Q.TRKKKR**	**. . . . . . . . .F. . . .**
**It004.2017**	**. . .D. . . . . . . . .D . . .**	**RH. . . .E.QR--.NKT. .V. . . .-.E**	**A. .V.Q.TRKK.R**	**. . . . . . . . .F. . . .**

**Fig 1:**
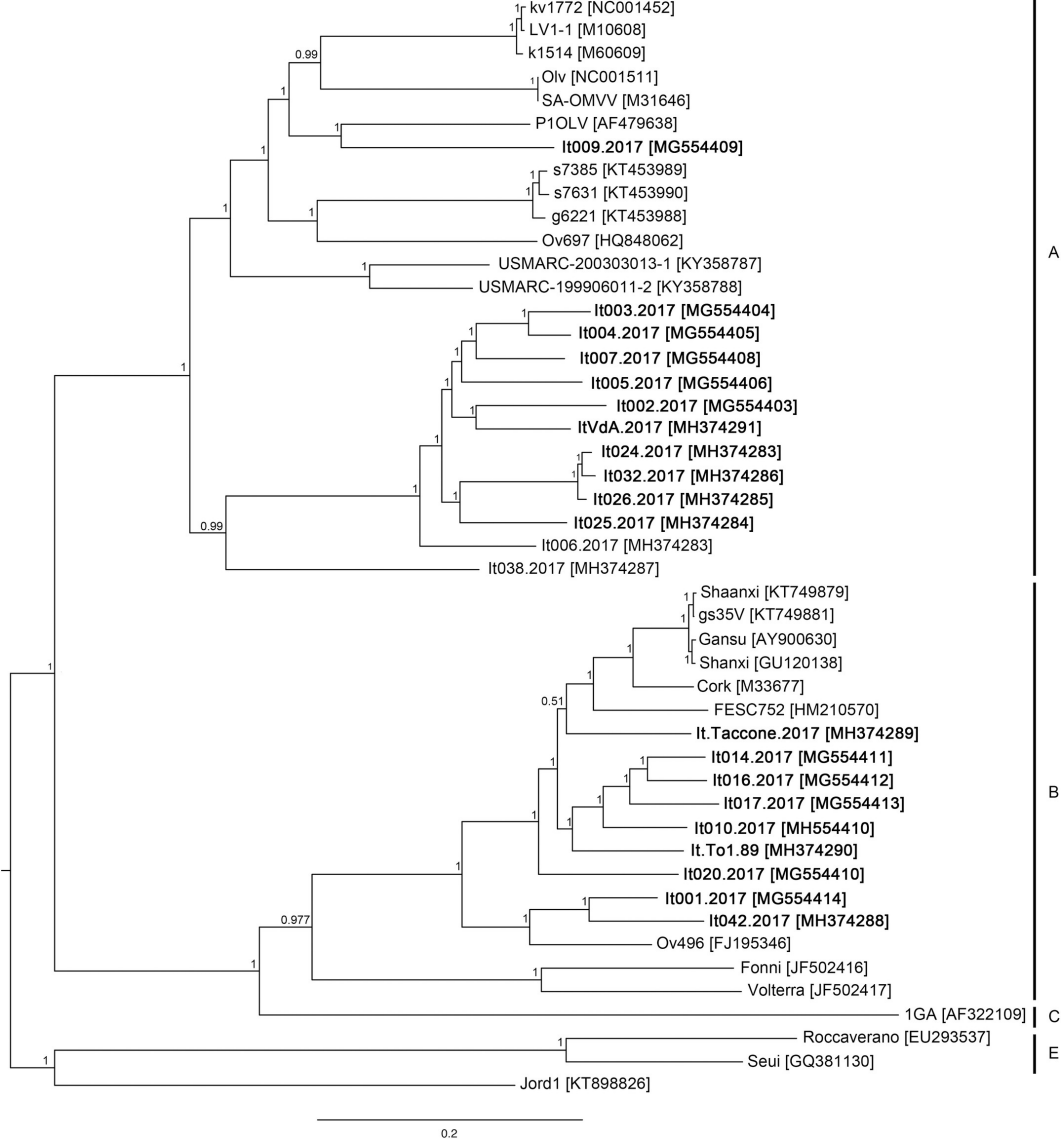
Bayesian tree based on the full genome sequences alignment. Newly characterized isolates are reported in bold. SRLV genotypes are reported. Posterior probability of each node is showed above branches.

**Fig 2:**
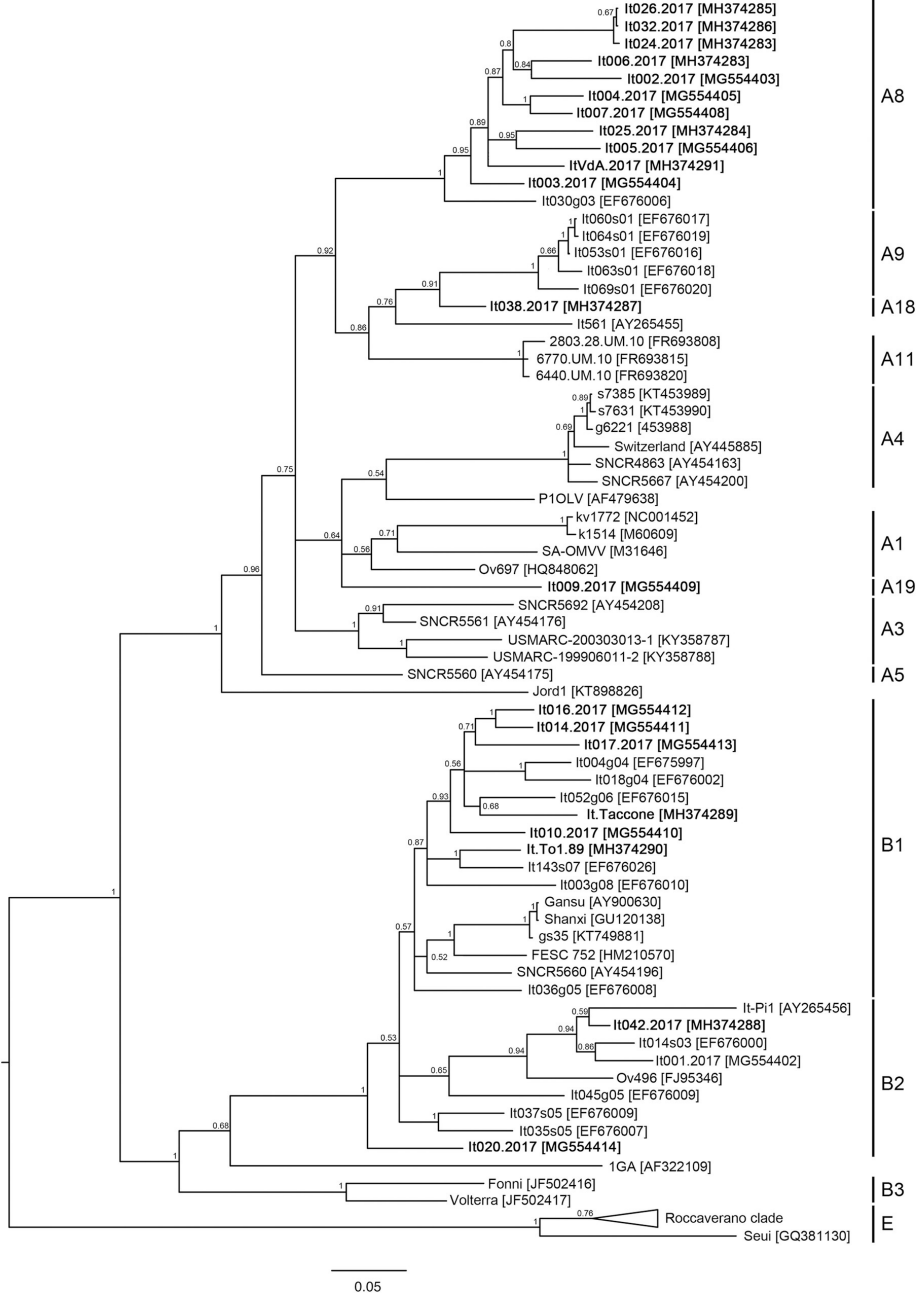
Bayesian tree based on the partial gag sequence alignment. Newly characterized isolates are reported in bold. SRLV subtypes are reported. Posterior probability of each node is showed above branches.

Phylogenetic trees clearly confirmed the genotype and the subtypes clustering of the new isolates considering both the complete genome (Fig 1) and partial *gag* gene sequence (Fig 2) alignments.

Full genome sequences of newly assembled isolates are available on GenBank (MG554402-MG554414 and MH374283-MH374291).

## Discussion

In this study, a new approach was developed addressing the full genetic characterization of SRLV isolates by NGS technologies. Since 1985, when the full genome sequence of the first isolate was published, a number of full genomes has been deposited, representing the prototype strains associated to specific diseases of relevant genotypes. These first genetic data represented a useful hallmark for SRLV diagnosis and control, opening new perspectives for recombinant antigen technologies and development of sensitive serological tests, covering the heterogeneity of lentiviruses in both sheep and goats. Moreover, genetic alignment has been progressively improved (in terms of quality and quantity) allowing the design of primer sets facilitating the amplification of at least different conserved genome regions. To date 17 subtypes of genotype A [11], 4 subtypes of genotype B [12], and two subtypes of genotypes C [13] and E [4,14] have been identified using standard end point partial amplification of the LTR and *gag*, *pol* and *env* genes followed by Sanger sequencing. This has led to a significant improvement of the knowledge on viral epidemiology, pathogenesis, diagnosis and control.

The method developed in this study allowed the characterization of 22 SRLV full genomes from fresh isolates, providing a fast and economically feasible tool for SRLV investigation. The availability of full genome, along with a viral isolate, is often necessary to evaluate antigenic and biological properties in detail. Moreover, the sequence coverage obtained with the described procedure supports the evidence of high heterogeneity among the isolates and within each genotype, as reported in previous studies [1,6].

According to *de novo* sequencing strategy potentially novel highly divergent genomes can be virtually detected, even in the presence of mixed infections, providing the genetic bases for the development of specific diagnostic tools. In this context, results presented in the study are quite intriguing and can be extrapolated to other known infected regions or to unexplored populations even if they are representative of a limited geographic area. This approach also allowed the genetic characterization of hypervariable regions such as HV3-5 within the *env* gene that may be difficult to amplify by conventional PCR due to strain specificity [15,16].

Keeping in mind the biological constraint of SRLV in the asymptomatic stage, the success of virus isolation highly depends on the viral load present in explant cultures from the main sites of viral persistence. According to previous experiences, spleen tissue gave the higher rate of success, followed by mammary gland and peripheral blood mononuclear cells (PBMC) co-culture [17]. Spleen *ex vivo* biopsy by needle aspiration produced enough red pulp fragments with the desired size that rapidly established a culture of terminally differentiated macrophages, possibly derived by red pulp resident cells. This explains the high proportion of viral strains obtained in the first passage. Contamination of fibroblastic-like culture is a normal feature in many tissue explants and spleen is not an exception. Terminally differentiated macrophages and dendritic cells are often replaced by overgrowing fibroblastic cells during cell culture propagation. In many instances, this results in adaptation of SRLV isolates able to infect fibroblasts and producing the characteristic cell fusion. However, this phenomenon is not always observed. The prototype strain Roccaverano was firstly isolated from spleen and mammary gland explants. Adaptation to standard foetal cells (synovial membrane, lung or choroid plexus) was unsuccessful [18] presumably due to restricted macrophage tropism [5]. About half of the strains isolated in the present study belonging to the genotype A did not show cell membrane fusion and a subset of them demonstrated impaired adaptation to overgrowing fibroblasts, as RT activity trended to decrease over time, as soon as infected macrophages died and were replaced by fibroblasts. This behavior is in agreement with a previous study on low pathogenic SRLV strains [19]. Since viral microevolution seems to be essential to drive tissue compartmentalization [16], we cannot exclude that viral isolation in asymptomatic animals may be restricted to canonical cell types compared with isolation from lentivirus specific gross lesions (i.e. lung, udder, synovial membrane).

However, in the present study all strains belonging to subtype B1 in goats and B2 in sheep were fusogenic *in vitro*, suggesting enhanced ability of the latter subtypes to efficiently replicate in spleen-derived fibroblasts. The first virological survey in the same area was carried out in the early nineties and later genetically characterized [2]. All strains were isolated from gross lesions and belonged to genotype B and now referred as B1 in goats and B2 in sheep [1]. More than 20 years later, the majority of goat isolates from asymptomatic animals belong to subtype A8; this finding strongly suggests that subtype A8 may represent a low pathogenic subtype that may have passed inadvertently causing persistent infections. Flock owners were not aware of any of the clinical signs attributable to SRLV before the French breeds entered the population in the early 1980s, further supporting this hypothesis. It should be noted that in the sampled area no control measures have been ever implemented and the great number of A8 isolates is unlikely to have emerged as a consequence of diagnostic escape, as happened for subtype A4 in Swiss goats [19,20]. In the latter experience, the poor performance of serological test in detecting SRLV A4 subtypes, most likely favored the spread of genotype A4 in goats, although the same test was quite effective in genotype B eradication campaigns (subtype B1).

Interestingly, the A4 subtype in Swiss goat is probably endemic in some Swiss regions but no SRLV-induced pathology has been recorded in Switzerland in the last 15 years [20]. A similar picture could be attributable to subtype A8 in northwest Italy.

It is noteworthy to consider the amino acid sequence spanning the hypervariable regions of the envelope protein (HV1 and HV2) which are believed to play a crucial role in cellular receptor binding. Interestingly, the A8 fusogenic strain contained an insertion of 2 residues within the HV2 motif and a greater proportion of basic amino acids, mimicking the prototype fusogenic strain A1 (Table 3). On the other hand, the sequence analysis of U3 region of the long terminal repeats was similar in A8 subgroup, being the transcription factor binding sites well conserved among isolates. These data taken together suggest that receptor binding, rather than transcription factors, may be associated to low pathogenic potential (Table 3) [21].

A certain degree of antigenic heterogeneity was observed in some isolates of subtype A8 and this may influence the sensitivity of some diagnostic tests. Sequence analysis of the immunodominant transmembrane epitope [22] revealed a non-synonymous mutation Y to F within the loop of disulfide bond (Table 2). In addition, the above mentioned P231Q mutation within the major capsid antigen was sufficient to drive the serotyping reactivity versus genotype E antigen leading to misclassification by standard ELISA tests. Since the genotype E derived antigen is usually not included in commercially available antigens, this A8 variant may potentially escape from standard serological test.

Only one goat showed an A8 virological positive outcome in the absence of serological reaction. The genome analysis of this isolate did not reveal any atypical epitope signature which may explain serological misdiagnosis. We cannot exclude a very recent infection in the window in which antibody response is not yet detectable or, instead, an impaired antibody production in late infection steps [23].

Among the sequences described in the present study, two possible new subtypes within genotype A were found (It009.2017 and It038.2017). Both were fusogenic *in vitro* (Table 1) and showed highest reactivity against genotype A-derived antigen in the genotyping ELISA test. In both cases, the new isolates did not show significant similarity with the available sequence data set (similarity values lower than the 85%). The former isolate is similar to A4 and A1 subtypes, belonging to the same monophyletic clade within the *gag* gene-based tree. The latter isolate is phylogenetically related to the sheep strain It-561 isolated in Tuscany in 1995 and to the A9 SRLV subtype (Fig 2). The structure of these clades suggests that the new isolates belong to different evolutionary lineages compared to the reference subtypes, but their position within the clade highlights the very high heterogeneity of SRLV, especially within the genotype A. This result shows how the SRLV population structure is complex and its evolutionary patterns are still largely unknown. The two different host species are characterized by different farming management and population sizes; this may influence the animal-pathogen interactions and consequently may drive the SRLV to evolve in different manners in different behaviors. Given the improvement of diagnostic and viral characterization tools, these results may help to understand the complexity of SRLV viral heterogeneity and should lead to consider an update in SRLV classification, considering both genetic and *in vitro* properties of the new isolates.

Moreover, the pathogenic potential of these less frequent subgroups (i.e. A18 and A19) will require additional studies since the serological tools are not fully able to differentiate the strains within each genotype.

In conclusion the proposed approach, involving virus purification from spleen biopsy followed by NGS, allowed the isolation and full genome characterization of 22 novel SRLV strains. The success of this method is based on the following features: i) spleen is one of the main target organs for SRLV persistence; ii) red pulp is a reserve of resident macrophages, the main target for SRLV replication *in vivo*; iii) RT activity is a sensitive and specific assay for revealing SRLV grown in cell culture; iv) de novo sequencing and assembling do not require previous genetic knowledge. Even if further studies are needed in order to validate the method and to asses its diagnostic performances we were able to detect both pathogenic and non pathogenic viral strains in goats and sheep, despite the limited sampling area, increasing the knowledge about SRLV genetic diversity.
